# Dietary isoleucine improved flesh quality, muscle antioxidant capacity, and muscle growth associated with AKT/TOR/S6K1 and AKT/FOXO3a signaling in hybrid bagrid catfish (*Pelteobagrus vachelli♀ × Leiocassis longirostris♂*)

**DOI:** 10.1186/s40104-021-00572-4

**Published:** 2021-04-19

**Authors:** Qin Jiang, Mingyao Yan, Ye Zhao, Xiaoqiu Zhou, Long Yin, Lin Feng, Yang Liu, Weidan Jiang, Pei Wu, Yan Wang, Defang Chen, Shiyong Yang, Xiaoli Huang, Jun Jiang

**Affiliations:** 1 College of Animal Science and Technology, Sichuan Agricultural University, Chengdu, 611130 China; 2 Animal Nutrition Institute, Sichuan Agricultural University, Chengdu, 611130 China; 3 Fish Nutrition and Safety Production University Key Laboratory of Sichuan Province, Sichuan Agricultural University, Ya’an, 625014 China

**Keywords:** Antioxidant capacity, Flesh quality, Hybrid bagrid catfish, Isoleucine, Myogenesis, Protein deposition

## Abstract

**Background:**

Muscle is the complex and heterogeneous tissue, which comprises the primary edible part of the trunk of fish and mammals. Previous studies have shown that dietary isoleucine (Ile) exerts beneficial effects on growth in aquatic animals. However, there were limited studies regarding the benefits of Ile on fish muscle and their effects on flesh quality and muscle growth. Thus, this study was conducted to explore whether dietary Ile had affected flesh quality and muscle growth in hybrid bagrid catfish (*Pelteobagrus vachelli♀ × Leiocassis longirostris♂*).

**Methods:**

A total of 630 hybrid fish, with an initial average body weight of 33.11 ± 0.09 g, were randomly allotted into seven experimental groups with three replicates each, and respectively fed seven diets with 5.0, 7.5, 10.0, 12.5, 15.0, 17.5, and 20.0 g Ile/kg diets for 8 weeks.

**Results:**

In the present study, we demonstrated that Ile significantly: (1) increased muscle protein and lipid contents and the frequency distribution of myofibers with ≤ 20 μm and ≥ 50 μm of diameter; (2) improved pH value, shear force, cathepsin B and L activities, hydroxyproline content, resilience, cohesiveness, and decreased cooking loss, lactate content, hardness, springiness, gumminess, and chewiness; (3) decreased reactive oxygen species (ROS), malondialdehyde (MDA), and protein carbonyl (PC) contents, *GCLC* and *Keap1* mRNA levels, and up-regulated *CuZnSOD*, *CAT*, *GPX1a, GST*, and *Nrf2* mRNA levels; (4) up-regulated the insulin-like growth factor 1, 2 (*IGF-1*, *IGF-2*), insulin-like growth factor 1 receptor (*IGF-1R*), proliferating cell nuclear antigen (*PCNA*), *Myf5*, *Myod*, *Myog*, *Mrf4*, and *MyHC* mRNA levels, and decreased *MSTN* mRNA level; (5) increased muscle protein deposition by activating AKT-TOR-S6K1 and AKT-FOXO3a signaling pathways.

**Conclusion:**

These results revealed that dietary Ile improved flesh quality, which might be due to increasing nutritional content, physicochemical, texture parameters, and antioxidant ability; promoting muscle growth by affecting myocytes hyperplasia and hypertrophy, and muscle protein deposition associated with protein synthesis and degradation signaling pathways. Finally, the quadratic regression analysis of chewiness, ROS, and protein contents against dietary Ile levels suggested that the optimal dietary Ile levels for hybrid bagrid catfish was estimated to be 14.19, 12.36, and 12.78 g/kg diet, corresponding to 36.59, 31.87, and 32.96 g/kg dietary protein, respectively.

**Supplementary Information:**

The online version contains supplementary material available at 10.1186/s40104-021-00572-4.

## Introduction

Recently, the global aquaculture production has reached about 82 million tons in 2018, and accounts for more than 50% of the worldwide fish food supply [1]. However, with the improvement of consumers’ health awareness, people pay more and more attention to the quality and nutritional composition of meat [2]. Thus, the aquaculture industry seems to be a growing interest in concerning about fish quality, which is influenced by a wide variety of external factors, such as feeding management, dietary composition, and amino acids ration level [3–6]. In fish, flesh quality is also regulated by nutritional factors, especially amino acids [7, 8]. As a member of branched-chain amino acids (BCAA), isoleucine (Ile) is not only the building block of protein but also the essential nutrient in aquafeeds, which plays a vital role in growth and flesh quality [9]. Our previous study have indicated that dietary Ile could improve the growth performance [10] and enhance the intestinal mucosal and physical barrier functions of hybrid bagrid catfish [11]. However, the effects of dietary Ile on flesh quality of hybrid bagrid catfish has not been extensively elucidated.

Fish flesh quality is affected by multifarious aspects including nutritional content, physicochemical, and texture parameters [12, 13]. The nutritional content was mainly reflected by muscle protein and lipid contents. Emerging shreds of evidence have shown that dietary Ile could increase muscle protein and lipid contents of grass carp (*Ctenopharyngodon idella*) [9] and fingerling spotted snakehead (*Channa punctatus*) [14]. These results provided possible evidence that dietary Ile could affect muscle nutritional content and merit further study. Additionally, physicochemical (pH value, water holding capacity, lactate, cathepsin, and hydroxyproline content) and texture parameters (hardness, springiness, cohesiveness, gumminess, chewiness, and resilience) are also the important indices for flesh quality [15]. Previous study have demonstrated that diet had a potential benefit to improve muscle flesh quality by regulating the physicochemical parameters of grass carp [9]. However, knowledge about the effects of dietary Ile on muscle texture parameters is scarce, which deserves further research. Some studies also have shown that flesh quality was negatively linked to the serious oxidative stress in carp [3, 9, 16]. Oxidative damage was primarily caused by excessive accumulation of reactive oxygen species (ROS), which could be effectively eliminated by the antioxidant system in fish [3, 8]. Our previous study have found that Ile could improve intestine function by increasing antioxidant ability via Kelch-like ECH-associated protein 1 (Keap1)- nuclear factor erythroid 2-related factor 2 (Nrf2) signaling pathway in hybrid bagrid catfish [10]. However, it remains unknown whether dietary Ile could improve the quality of flesh by regulating Keap1-Nrf2 signaling pathway in hybrid bagrid catfish.

The muscle is the complex and heterogeneous tissue, which comprises the primary edible part of the trunk of fish [17]. Fish muscle growth is a dynamic process involving both the hyperplasia of myofibers and hypertrophy of existing myofibers [18], which is under the control of a series of transcription factors such as insulin-like growth factors (IGFs), proliferating cell nuclear antigen (PCNA), myogenic regulatory factors (MRFs), and myostatin (MSTN) [19, 20]. The IGFs, including IGF-1 and IGF-2, play a pivotal role in the growth and functional maturation of fish muscle [21]. Previous studies have revealed that IGFs could improve muscle growth of Japanese amberjack (*Seriola quinqueradiata*) [22], hybrid bagrid catfish [23], and gilthead sea bream (*Sparus aurata*) [24]. The MRFs, including myogenic determination (Myod), myogenic factor 5 (Myf5), myogenic regulatory factor 4 (Mrf4), and myogenin (Myog), play a critical role in myoblast proliferation and myogenic differentiation [21]. The MSTN acts as a negative regulator of muscle growth by inhibiting the myogenesis in fish [25, 26]. Despite muscle growth induced by these factors have reported in gilthead sea bream, rainbow trout (*Oncorhynchus mykiss*), and zebrafish (*Danio rerio*) [24, 27, 28]. Several studies in our laboratory also have reported that dietary threonine [23] and leucine [29] could promote the muscle growth of hybrid bagrid catfish by regulating these factors. However, there was unclear whether dietary Ile could improve muscle growth through impacting hyperplasia and hypertrophy in fish.

Fish muscle growth primarily associated with protein deposition of muscle [30]. Muscle protein deposition is the result of a balance between protein synthesis and protein degradation. The IGF-1 and IGF-2 mediate muscle growth by binding to IGF-1 receptor (IGF-1R) at the cell membrane [31, 32]. Several studies have demonstrated that IGF-1 promotes muscle growth via the IGF-1R- phosphoinositide 3-kinase (PI3K)- protein kinase B (AKT) pathway [32–34]. As a response element of the PI3K-AKT pathway, target of rapamycin (TOR) acts a central role in cell growth, development, and metabolism. The TOR could promote protein synthesis by phosphorylating ribosomal protein S6 kinase 1 (S6K1) and the eukaryotic translation initiation factor 4E (eIF4E) binding protein 1 (4E-BP1) in fish [35, 36]. Previous studies have reported that Ile could activate TOR signaling pathway and regulate the phosphorylation of S6K1 and 4E-BP1 in bovine [37] and goat [38] mammary tissue, ultimately stimulating protein synthesis. Gan et al. [9] have reported that dietary Ile increased the mRNA expressions of *TOR* and *S6K1* in grass carp muscle. On the other hand, protein synthesis and degradation pathways are interlinked in muscle [39]. The PI3K-AKT signaling pathway also regulates protein degradation by activating the forkhead box protein O3a (FOXO3a) and subsequently inhibiting the ubiquitin-proteasome system (UPS), which mediates 80–90% protein degradation [40, 41]. The UPS associated with two key ubiquitin ligases (muscle atrophy F box, MAFBX and muscle-specific RING finger 1, MURF-1) are responsible for specific induction of the muscle protein degradation [42–44]. In previous studies, BCAA decreased amount of MURF-1 and MAFBX during proliferation and differentiation in mice myocytes [45] and protein levels of MURF-1 and MAFBX in piglet muscle [46]. It has been well reported that dietary leucine could regulate protein synthesis and degradation through targeting the corresponding signaling pathways in fish muscle [29]. However, whether dietary Ile could affect protein synthesis and protein degradation involving IGF-1-AKT-TOR and IGF-1-AKT-FOXO3a signaling pathways in fish muscle remain unknown.

Yellow catfish (*Pelteobagrus fulvidraco*) is a very extensive and commercially important freshwater fish species farmed in China [47]. Recently, our laboratory began to breed hybrid bagrid catfish (*Pelteobagrus vachelli♀ × Leiocassis longirostris♂*) in 2017, and optimal dietary threonine [23] and leucine [29] levels based on the optimal muscle growth have been investigated. This study used the same growth trial from our previous study, which have determined the effects of dietary Ile on fish growth performance [10]. The present study was performed to explore the effects of dietary Ile on flesh quality, muscle growth, protein synthesis- and degradation-related signaling pathways in hybrid bagrid catfish.

## Materials and methods

### Experimental diets and design

All experimental conditions and protocols were approved by the Animal Ethic Advisory Committee of Sichuan Agricultural University under permit No. DKY-S20170512. The proximate composition of the basal diet is given in the additional files (Table S1), which is the same as our previous study [10]. Fish meal, soybean meal, rapeseed meal, and corn gluten meal were used as the primary protein sources. Soybean oil and wheat flour were used as the main lipid and starch sources. Dietary crude protein and lipid were fixed at 387.8 and 71.0 g/kg diets respectively. The Ile concentrations of seven experimental diets were 5.0 (control diet), 7.5, 10.0, 12.5, 15.0, 17.5, and 20.0 g/kg diet. All diets were made iso-nitrogenous with the addition of appropriate amounts of glycine. After thoroughly mixing all diet ingredients, water was added to achieve a proper pelleting consistency and pelleted mixture through the screw Extruder (SYX62, Xiamen, China) at 2-mm diameter. The processing conditions were as follows: 100 r/min screw speed, 127 °C temperature, and 30–45 atm pressure. Floating extruded pellets were air-dried and then stored at − 20 °C in plastic bags until used.

### Fish feeding and management

Hybrid bargid catfish were purchased from Rong Sen Corporation (Sichuan, China). Fish were adapted to the experimental environment for 4 weeks. A total of 630 fish of the approximate size with an average initial weight of 33.11 ± 0.09 g, were randomly allocated into 21 concrete tanks (210 L; 2.00 m × 1.00 m × 1.05 m), resulting in 30 fish each tank. During the 8-week trial, fish were fed with their respective diets to apparent satiation two times (8:00 and 18:00) under natural light and dark cycle. The water quality, temperature, dissolved oxygen, pH, nitrite, and ammonia content were recorded throughout the experiment (25 ± 2 °C, 5 mg/L, 7.0 ± 0.3, < 0.05 mg/L, < 0.5 mg/L). Continuous flowing water was maintained the rate of 1.2 L/min each tank.

### Sample collection and analysis

After a fasting period of 24 h, fish in each tank were counted and weighed at the termination of the feeding trial. Then, 12 fish from each tank were randomly selected and anaesthetized in 50 mg/L benzocaine tub [48]. Muscle samples from the left sides of 6 fish in each tank were taken and kept frozen (− 20 °C) for muscle composition analysis. Meanwhile, from the right sides of the same fish, muscle samples were obtained determination of physiochemical- and antioxidant-related parameters. The muscle samples from the left sides of another 6 fish were frozen in liquid nitrogen, and then stored at − 80 °C for biochemical parameters and molecular analysis [49]. The muscle from the right sides of same 4 fish were cut into two parts of 1 cm × 1 cm × 1 cm muscle blocks using a mold, which were used for the determination of texture parameters [6]. The muscle from the right sides of another 2 fish were used for histological analysis. Although 12 fish from each treatment were sampled, 6 sampled fish from each tank were randomly selected for further analysis.

The moisture, ash, protein, and lipid contents of muscle were determined according to the previously described [50]. Muscle pH, shear force, and cooking loss were determined as previously mentioned [51]. The lactic acid content in the muscle was measured using commercially kits according to the manufacturer’s protocols (Nanjing Jiancheng Bioengineering Institute, Nanjing, China). The muscle hydroxyproline content was determined using the method reported by Periago et al. [17]. The activities of cathepsin B and L were measured using the procedure, according to Jiang et al. [3].

The muscle samples were homogenized in 10 volumes (mg/mL) of ice-cold physiological saline solution and centrifuged at 6000×*g* for 20 min at 4 °C, respectively. The supernatant was collected for enzyme activity analysis. The contents of protein, MDA, PC, GSH and the activities of T-SOD, CAT, GPX, GST, GR, ASA, and AHR were measured using commercial kits [49]. The ROS contents were measured as decreased by LeBel et al. [52].

The muscle samples with an average weight 4.6 ± 0.5 g were cut into 1.0-cm thick cuboids for texture profile analysis (TPA) measurements. The hardness, cohesiveness, springiness, gumminess, chewiness, and resilience were measured using the method described by Zhang et al. [53]. Then the probe was compressed twice with a constant moving speed of 1.0 mm/s and the deformation rate was set at 75% of the specimen. Textural parameters were collected and calculated by using a force-deformation curves [16].

### Histological analysis

Transverse white muscle sections (5 μm) were embedded in paraffin, and 6 fish per treatment (2 fish per tank) were stained with haematoxylin-eosin to evaluate muscle morphology [54]. Stained sections were visualized under an optic microscope and images were captured by a DS-Ril camera and software. Using Image Pro 6.0 software, myofiber number and diameter were measured for about 300 adjacent myofibers of every section each fish. Myofibers were divided into three diameter ranges (≤ 20 μm, 20–50 μm, or ≥ 50 μm) as described by de Almeida et al. [55]. Myofiber frequency was expressed as the number of myofibers in each type of diameter class relative to the total number of myofibers.

### Real-time polymerase chain reaction (RT-PCR) analysis

Total RNA was extracted using RNAiso Plus (Takara, Dalian, China) and the integrity and quality of RNA were determined by 1.5% agar-gel electrophoresis and spectrophotometry, respectively. The PrimeScript™ RT reagent Kit (Takara, Dalian, China) was used to synthesize the complementary DNA (cDNA) from 1 μg RNA. The quantification of selected genes transcript levels was performed via the real-time quantitative PCR. The real-time PCR conditions were as follows: 94 °C for 5 min, followed by 40 cycles at 95 °C for 5 s, annealing at a different temperature for each gene for 10 s and 72 °C for 15 s. In order to verify the specificity of each amplicon, melting curve analysis was drawn at 65–95 °C with reading once per 0.5 °C and performed after amplification. The amplification efficiency of genes, ranging from 95% to 105%, were calculated by specific gene standard curves generated from 10-fold serial dilutions. Specific primers of hybrid bagrid catfish were exhibited in the additional files (Table S2). The relative gene expression levels were evaluated using the 2^−ΔΔCt^ method according to Livak et al. [56], using *β-actin* and 18S rRNA as the internal control genes [57].

### Western blot analysis

Western blot was done to determine the muscle protein abundance following published methods [23]. Briefly, the proteins were extracted from muscle with RIPA buffer (Beyotime, Shanghai, China). The total 30 μg protein in lysates were separated by SDS-PAGE gel electrophoresis and transferred to a polyvinylidene fluoride (PVDF) membrane. The membrane was blocked with blocking buffer for 1 h at 20 °C, then incubated at 4 °C overnight with primary antibody. The primary antibodies used in this study were AKT (1:1000; total and Ser473, Cell Signaling Technology Inc., Danvers, MA, USA), TOR (1:2000; total and Ser2448, CST), S6K1 (1:2000; total and Thr421/Ser424, CST), FOXO3a (1:3000; total and Ser253, Abcam, Cambridge, MA, USA), MAFBX and MURF-1 (1:4000; Zen Biotechnology, Chengdu, Sichuan, China). The β-actin (1:1000; CST) was used as the control protein. The blots were washed five times for 3 min each, followed by 2 h incubation with appropriate secondary antibody in TBST at 25 °C. The blots were visualized using an enhanced chemoluminescence (ECL) kit (Beyotime). This experiment was repeated at least in triplicate.

### Statistical analysis

All results were subjected to one-way ANOVA analysis followed by Tukey’s HSD test to evaluate the differences within treatments using the SPSS version 20.0 (SPSS Institute Inc., Chicago, USA). The trends of linear and quadratic analyses were conducted by using SAS software version 8.0 (SAS Institute Inc., North Carolina, USA). *P* < 0.05 were considered significant. Data were expressed as the mean ± SEM.

## Results

### Growth performance, muscle composition, and flesh physicochemical parameters

The present study used the same animal trail as our previous study and the results of graded levels of dietary Ile on growth parameters have been previously reported [10]. Briefly, there was no differences in survival rate and initial body weight among treatments (*P* > 0.05). The final body weight, feed intake, percent weight gain, specific growth rate, protein efficiency ratio, and feed efficiency were quadratically increased with dietary Ile level, and reached the maximum at 12.5 g/kg diet (*P <* 0.05) (Fig. S1). As shown in Tables 1 and 2, fish fed 12.5 g/kg diet increased muscle crude protein and lipid contents (*P <* 0.05). Fish fed 12.5 g/kg diet had lower moisture content than those fish fed other Ile-level diets (*P <* 0.05). However, there was no significant difference in ash value among treatments. Muscle shear force was the maximum for fish fed 12.5 g/kg diet and the minimum for fish fed 10.0 g/kg diet (*P <* 0.05). The pH_45min_, lactate, and hydroxyproline contents were increased with Ile levels increasing up to 12.5 g/kg diet and decreased thereafter (*P <* 0.05). Cathepsin B and L activities were significantly depressed with Ile levels increasing up to 10.0 g/kg diet and then gradually increased (*P <* 0.05). However, there were no significant differences in pH_24h_ and cooking loss among groups.

**Table 1:** Muscle composition of hybrid bagrid catfish fed experimental diets containing graded levels of Ile for 8 weeks

Items	Dietary Ile levels, g/kg							SEM	*P*-values	
	5.0	7.5	10.0	12.5	15.0	17.5	20.0		Linear	Quadratic
Moisture	74.82^a^	73.90^b^	72.22^d^	71.75^e^	73.49^c^	74.02^b^	73.99^b^	0.06	< 0.01	< 0.0001
Protein	16.89^a^	17.71^c^	18.59^d^	18.80^d^	17.67^c^	17.34^b^	17.35^b^	0.07	0.45	< 0.0001
Lipid	5.99^a^	6.14^b^	6.82^e^	7.06^f^	6.81^de^	6.68^cd^	6.57^c^	0.03	< 0.0001	< 0.0001
Ash	1.63	1.62	1.64	1.63	1.65	1.62	1.62	0.01	0.93	< 0.05

Values are means ± SEM of three replicate groups with 6 fish in each group. Values within the same rows having different superscripts are significantly different (*P <* 0.05)

**Table 2:** Muscle physiochemical parameters of hybrid bagrid catfish fed experimental diets containing graded levels of Ile for 8 weeks^1^

Items^2^	Dietary Ile levels, g/kg							SEM	*P*-values	
	5.0	7.5	10.0	12.5	15.0	17.5	20.0		Linear	Quadratic
pH_45min_	7.11^a^	7.15^a^	7.50^ab^	7.62^b^	7.60^b^	7.59^b^	7.50^ab^	0.05	< 0.0001	< 0.0001
pH_24h_	6.83	6.83	6.78	6.78	6.89	6.83	6.78	0.03	0.79	0.76
Shear force	0.26^abc^	0.22^a^	0.19^a^	0.32^c^	0.25^abc^	0.32^bc^	0.22^ab^	0.02	0.06	0.11
Cooking loss	20.49	18.27	19.78	19.28	19.42	17.45	19.25	0.72	0.16	0.56
Lactate	0.27^c^	0.24^bc^	0.21^abc^	0.16^a^	0.17^ab^	0.19^ab^	0.18^ab^	0.01	< 0.0001	< 0.01
Cathepsin B	2.04^abc^	1.72^ab^	1.63^a^	2.16^abc^	2.44^c^	3.34^d^	2.38^bc^	0.08	< 0.0001	0.12
Cathepsin L	0.87^c^	0.88^c^	1.05^d^	0.80^bc^	0.74^b^	0.72^b^	0.61^a^	0.01	< 0.0001	< 0.0001
Hydroxyproline	0.15^ab^	0.22^bc^	0.26^c^	0.49^d^	0.30^c^	0.13^ab^	0.12^a^	0.01	< 0.01	< 0.0001

^1^ Values are mean ± SEM of three replicate groups with 6 fish in each group. Mean values with the different superscripts in the same row are significantly different (*P <* 0.05)

^2^ Shear force, N/(mm·s); Cooking loss, %; Lactate content, mmol/g protein; Cathepsin B and L activities, U/g protein; Hydroxyproline concentration, μg/mg protein

### Muscle cellularity and texture parameters

As shown in Table 3, dietary Ile did influence the myofibers’ frequencies in the three ranges of myofiber diameters (≤ 20 μm; 20–50 μm; ≥ 50 μm). Frequency distribution of myofibers in types of fiber diameter ≤ 20 μm and ≥ 50 μm were increased with the increasing dietary Ile levels up to 12.5 g/kg diet and then decreased (Table 4, *P* < 0.05). The muscle hardness, gumminess, and chewiness decreased with dietary Ile levels increasing up to 12.5 g/kg diet and increased thereafter (Table 4, *P* < 0.05). Fish fed 12.5 g/kg diet had higher muscle cohesiveness and springiness than fish fed other diets (Table 4, *P* < 0.05). The muscle resilience (Table 4) increased with dietary Ile levels increasing to 10.0 g/kg diet (*P* < 0.05) and plateaued thereafter (*P* > 0.05).

**Table 3:** Muscle fibers cellularity, % frequency of hybrid bagrid catfish fed diets with graded levels of Ile for 8 weeks^1^

Items^2^	Dietary Ile levels, g/kg							SEM	*P*-values	
	5.0	7.5	10.0	12.5	15.0	17.5	20.0		Linear	Quadratic
d ≤ 20 μm	24.07^a^	26.18^ab^	25.12^ab^	33.12^c^	29.21^bc^	23.81^a^	24.04^a^	0.51	0.74	< 0.0001
20 < d < 50 μm	66.91^d^	63.42^cd^	58.69^bc^	48.93^a^	55.56^b^	64.31^cd^	66.42^d^	0.80	0.52	< 0.0001
d ≥ 50 μm	9.03^a^	10.40^a^	16.19^bc^	18.75^c^	15.24^b^	11.89^ab^	10.02^a^	0.41	< 0.05	< 0.0001

^1^ Values are means ± SEM of three replicate groups with 2 fish in each group. Mean values with the different superscripts in the same row are significantly different (*P <* 0.05)

^2^ d, diameter of muscle fibers

**Table 4:** Texture profile analysis properties of hybrid bagrid catfish fed experimental diets containing graded levels of Ile for 8 weeks^1^

Items^2^	Dietary Ile levels, g/kg							SEM	*P*-values	
	5.0	7.5	10.0	12.5	15.0	17.5	20.0		Linear	Quadratic
Hardness	1690.94^c^	1167.61^b^	960.48^ab^	745.61^a^	1121.86^b^	1053.46^ab^	1040.19^ab^	49.51	< 0.0001	< 0.0001
Springiness	0.39^ab^	0.43^b^	0.39^ab^	0.44^b^	0.36^a^	0.41^ab^	0.39^ab^	0.01	0.11	0.15
Cohesiveness	0.23^a^	0.28^bc^	0.30^bc^	0.31^c^	0.27^b^	0.28^bc^	0.30^bc^	0.01	< 0.01	< 0.05
Gumminess	384.49^b^	321.81^ab^	289.33^ab^	226.26^a^	300.96^ab^	290.92^ab^	311.7^ab^	13.51	< 0.01	< 0.0001
Chewiness	155.33^b^	140.09^ab^	115.81^ab^	98.36^a^	109.10^ab^	116.23^ab^	124.19^ab^	6.26	< 0.01	< 0.0001
Resilience	0.13^a^	0.15^a^	0.16^b^	0.15^b^	0.15^b^	0.15^b^	0.16^b^	0.01	0.18	0.39

^1^ Values are mean ± SEM of three replicate groups with 4 fish in each group. Mean values with the different superscripts in the same row are significantly different (*P <* 0.05)

^2^ Hardness, g; Springiness, cm; Gumminess, g; Chewiness, g

### Muscle antioxidant-related parameters

As indicated in Table 5, ROS, MDA, and PC contents decreased with increasing dietary Ile levels up to 12.5 g/kg diet and rose thereafter (*P <* 0.05). The GSH content and ASA, T-SOD, and CAT activities reached a maximum at 12.5 g/kg diet and decreased thereafter (*P <* 0.05). Fish fed 10.0 g/kg diet had higher AHR and GST activities than those fed other diets (*P <* 0.05). The GR activity elevated with increasing dietary Ile levels up to 15.0 g Ile/kg diet and then gradually decreased (*P <* 0.05). However, Ile did not affect GPX activity in fish muscle (*P* > 0.05).

**Table 5:** The contents of ROS, MDA, PC, and GSH, and the activities of ASA, AHR, T-SOD, CAT, GPX, GST, and GR in the muscle of hybrid bagrid catfish fed experimental diets containing graded levels of Ile for 8 weeks

Items	Dietary Ile level, g/kg							SEM	*P*-values	
	5.0	7.5	10.0	12.5	15.0	17.5	20.0		Linear	Quadratic
ROS, % DCF fluorescence	100.00^de^	81.84^c^	61.68^b^	37.79^a^	36.13^a^	86.07^cd^	113.90^e^	0.72	< 0.0001	< 0.0001
MDA, nmol/mg protein	3.31^b^	2.29^ab^	1.65^a^	1.56^a^	1.98^a^	2.50^ab^	5.47^c^	0.27	< 0.01	< 0.0001
PC, nmol/mg protein	6.53^c^	6.06^bc^	4.40^ab^	3.60^a^	4.05^a^	4.52^ab^	5.01^abc^	0.31	< 0.0001	< 0.0001
GSH, mg/g protein	2.07^a^	2.76^bc^	3.25^cd^	3.51^d^	3.11^cd^	2.86^bcd^	2.37^ab^	0.12	0.15	< 0.0001
ASA, U/mg protein	143.59^a^	181.50^b^	240.67^cd^	284.19^e^	245.60^d^	209.21^bc^	176.86^ab^	4.19	< 0.0001	< 0.0001
AHR, U/mg protein	93.45^a^	95.32^a^	184.63^e^	163.67^d^	146.11^c^	114.58^b^	102.72^ab^	2.06	< 0.05	< 0.0001
T-SOD, U/mg protein	39.35^ab^	41.11^ab^	43.89^b^	46.63^b^	41.34^ab^	39.27^ab^	34.11^a^	1.23	< 0.01	< 0.0001
CAT, U/mg protein	1.47^a^	2.49^b^	3.33^c^	3.21^c^	3.15^bc^	2.99^bc^	2.94^bc^	0.15	< 0.0001	< 0.0001
GPX, U/mg protein	34.08	36.12	38.32	40.19	43.72	41.62	40.31	1.84	< 0.01	0.06
GST, U/mg protein	96.58^bcd^	100.03^bcd^	108.74^d^	105.23^cd^	93.68^abc^	89.56^ab^	82.48^a^	1.81	< 0.0001	< 0.0001
GR, U/mg protein	5.21^a^	6.94^bc^	8.02^cd^	9.76^e^	9.86^e^	9.55^de^	5.62^ab^	0.27	< 0.0001	< 0.0001

Values are mean ± SEM of three replicate groups with 6 fish in each group. Mean values with the different superscripts in the same row are significantly different (*P <* 0.05)

### Muscle antioxidant related genes and signaling molecules expression

**Fig. 1:**
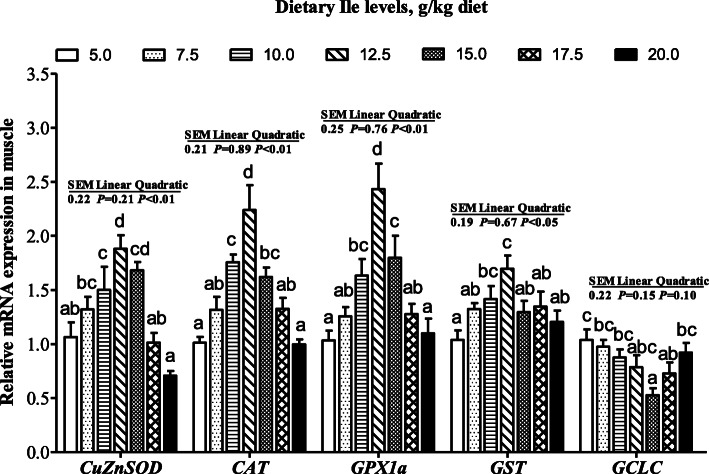
The mRNA levels of *CuZnSOD*, *CAT*, *GST*, *GPX1a*, and *GCLC* in muscle of hybrid bagrid catfish fed diets containing graded levels of Ile. Data represent means ± SEM, of six replicates. Values having different letters are significantly different (*P* < 0.05)

**Fig. 2:**
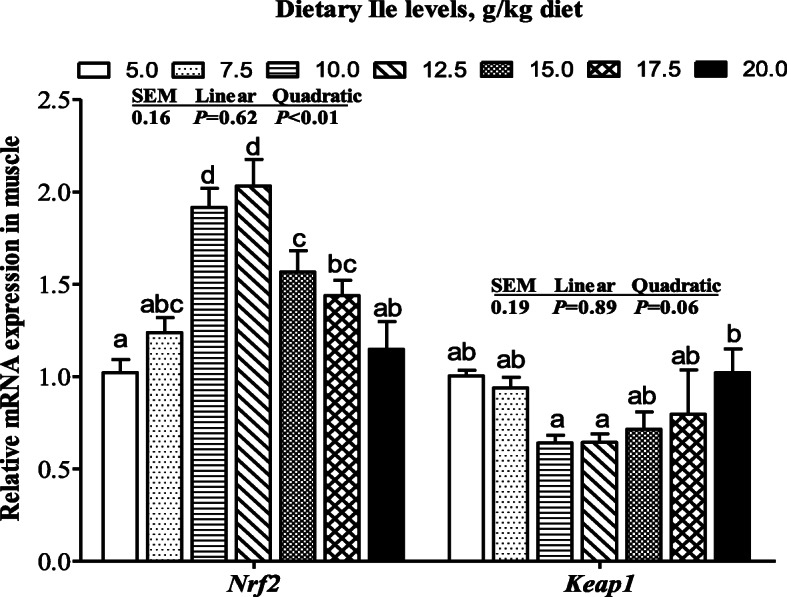
The mRNA levels of *Nrf2* and *Keap1* in muscle of hybrid bagrid catfish fed diets containing graded levels of Ile. Data represent means ± SEM, of six replicates. Values having different letters are significantly different (*P* < 0.05)

The mRNA levels of *CuZnSOD*, *CAT*, *GPX1a*, *GST*, and *GCLC* were presented in Fig. 1. The *CuZnSOD*, *CAT*, and *GPX1a* mRNA levels increased with dietary Ile levels increasing up to 12.5 g/kg diet and decreased thereafter (*P* < 0.05). The *GST* mRNA level increased with dietary Ile levels increasing up to 12.5 g/kg diet and plateaued thereafter (*P* < 0.05). The *GCLC* mRNA level showed a gradually decreased trend from 5.0 to 15.0 g/kg diet and then increased (*P* <  0.05). As shown in Fig. 2, *Nrf2* mRNA level increased with dietary Ile levels increasing up to 12.5 g/kg diet and decreased thereafter and *Keap1* mRNA levels exhibited the opposite pattern to *Nrf2* (*P* < 0.05).

### Muscle growth related genes and signaling molecules expression

**Fig. 3:**
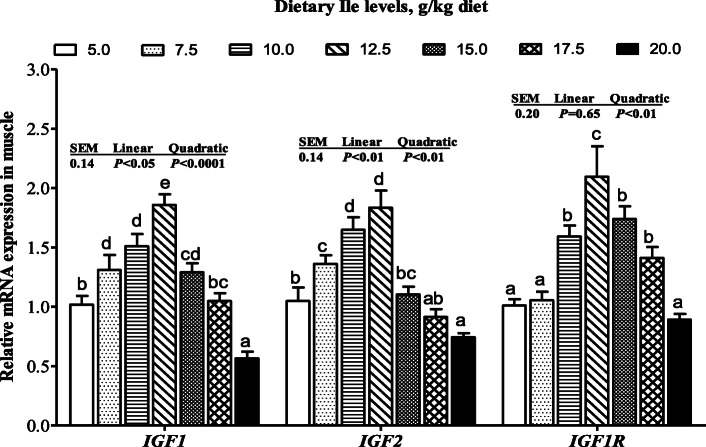
The mRNA levels of *IGF1*, *IGF2*, and *IGF1R* in muscle of hybrid bagrid catfish fed diets containing graded levels of Ile. Values are means ± SEM, of three replicates, with six fish in each replicate, and different letter denotes significant difference (*P* < 0.05)

**Fig. 4:**
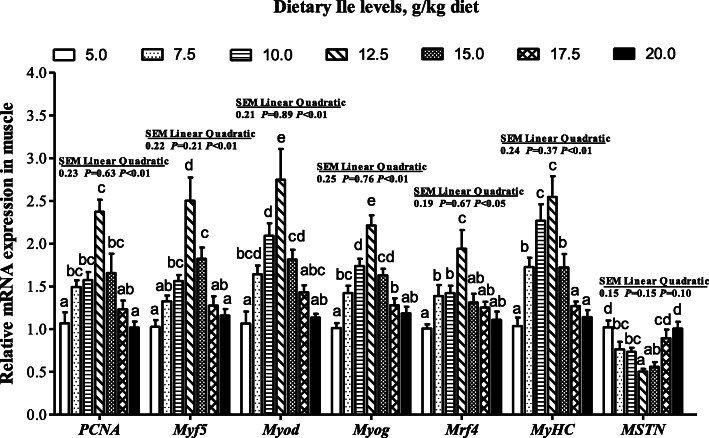
Effects of dietary Ile on muscle growth related gene mRNA levels in muscle of hybrid bagrid catfish fed diets containing graded levels of Ile. Values are means ± SEM, of three replicates, with six fish in each replicate, and different letter denotes significant difference (*P* < 0.05)

The *IGF1*, *IGF2*, and *IGF1R* mRNA levels increased with dietary Ile levels increasing up to 12.5 g/kg diet and then decreased (Fig. 3, *P <* 0.05). As presented in Fig. 4, mRNA levels of *PCNA*, *Myf5*, *Myod*, *Myog*, *Mrf4*, and *MyHC* significantly increased with Ile levels increasing up to 12.5 g/kg diet and decreased thereafter. However, the *MSTN* mRNA level showed an opposite trend of *MyHC* (Fig. 4, *P <* 0.05).

### Muscle protein synthesis related genes expressions and AKT-TOR-S6K1 signaling activity

**Fig. 5:**
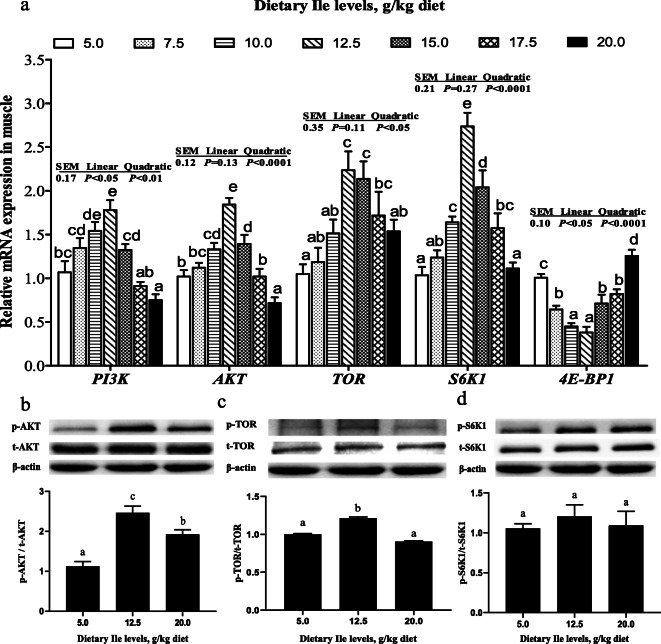
Effect of dietary Ile on the PI3K-AKT-TOR signaling pathway in hybrid bagrid catfish muscle fed diets containing graded levels of Ile. (**a**) The mRNA levels of *PI3K*, *AKT*, *TOR*, *S6K1*, and *4E-BP1*; (**b**) Protein abundances of p-AKT (Ser473), AKT, and ratio of p-AKT and AKT; (**c**) Protein abundances of p-TOR (Ser2448), TOR, and ratio of p-TOR and TOR; (**d**) Protein abundances of p-S6K1 (Thr421/Ser424), S6K1, and ratio of p-S6K1 and S6K1. Data represent means ± SEM, of three replicates. Values having different letters are significantly different (*P* < 0.05)

As shown in Fig. 5a, *PI3K*, *AKT*, *TOR*, and *S6K1* mRNA levels increased with Ile levels increasing to 12.5 g/kg diet and then decreased (*P* < 0.05). Conversely, *4E-BP1* mRNA level showed an opposite trend of *S6K1* (Fig. 5a, *P <* 0.05). Fish fed with 12.5 g/kg diet had elevated p-AKT protein abundance in the muscle (Fig. 5b, *P* < 0.05), and no significant differences was observed in total AKT protein abundance. Compared with both 5.0 and 20.0 g/kg diets, fish fed 12.5 g/kg diet exhibited a higher level of p-TOR/TOR (Fig. 5c, *P* < 0.05). Fish fed a diet containing 12.5 g/kg diet had higher p-S6K1 protein abundance (Fig. 5d) and did not alter total S6K1 protein abundance (*P* > 0.05).

### Muscle protein degradation related genes expressions and AKT-FOXO3a signaling activity

**Fig. 6:**
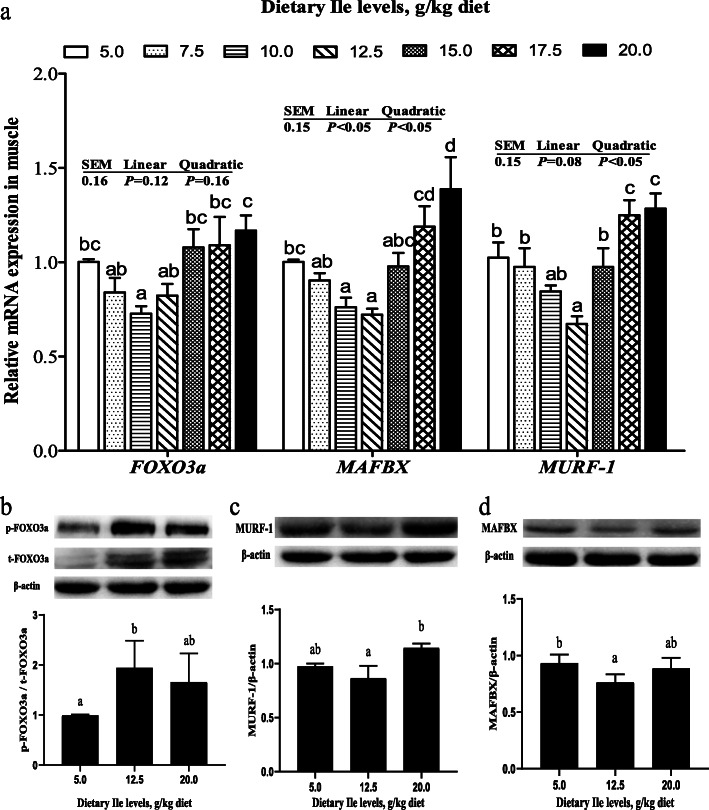
Effects of dietary Ile on the protein degradation related signaling pathway in hybrid bagrid catfish muscle fed diets containing graded levels of Ile. (**a**) The mRNA levels of *FOXO3a*, *MAFBX*, and *MURF-1*; (**b**) Protein abundances of p-FOXO3a (Ser253), FOXO3a, and ratio of p-FOXO3a and FOXO3a; Protein abundances of MURF-1 (**c**) and MAFBX (**d**). Data represent means ± SEM, of three replicates. Values having different letters are significantly different (*P* < 0.05)

The *FOXO3a* mRNA levels of fish fed with 7.5, 10.0, and 12.5 g/kg diets were lower than those fed with other diets (Fig. 6a, *P <* 0.05). The *MAFBX* and *MURF-1* mRNA levels decreased with dietary Ile levels increasing up to 12.5 g/kg diet and increased thereafter (Fig. 6a, *P* < 0.05). Fish fed with 12.5 g/kg diet had elevated p-FOXO3a and total FOXO3a protein abundances (Fig. 6b, *P* < 0.05). Compared with both 5.0 and 20.0 g/kg diets, fish fed 12.5 g/kg diet exhibited lower abundances of MURF-1 (Fig. 6c) and MAFBX (Fig. 6d).

## Discussion

This study used the same growth trail as our previous study, which showed that dietary Ile significantly improved the growth performance of hybrid bagrid catfish [10]. Fish growth is mainly determined by growth and protein deposition of muscle, which is the central edible portion for consumers [17]. With the enhancement of the living standard and the gradual enhancement of health awareness, healthy meat consumption would become the mainstream. Therefore, we seek to explore the potential benefits of dietary Ile on flesh quality and muscle growth.

### Dietary Ile improved fish flesh quality

Fish flesh quality can be partly reflected by the nutrient composition of muscle, which is perceived by the consumers [50, 58]. Muscle protein and lipid contents represent the principal nutritional value of flesh. In the present study, fish fed 12.5 g/kg diet increased muscle crude protein and lipid contents, indicating that dietary Ile could improve the flesh nutritional quality. Previous studies have shown that dietary Ile could significantly increase muscle crude protein and fat contents in grass carp [9] and Nile tilapia (*Oreochromis niloticus*) [59]. Besides, flesh quality is also strongly regulated by physicochemical and texture characteristics, which are the most important aspects of attracting approach to consumers [60]. The pH value is a key factor affecting the conversion of muscle to meat [13]. The flesh pH always decreases after slaughter mainly due to the accumulation of lactic acid [61]. In the present study, dietary Ile could increase pH_45min_ value and reduce the lactate content of fish muscle. A similar result was reported in grass carp [9]. The increased fillet juiciness was attributed to the decreased cooking loss. In this study, dietary Ile decreased the cooking loss of fish muscle. Previous studies have shown that cathepsin activities could affect fillet juiciness and firmness in Atlantic salmon (*Salmo salar*) [62] and grass carp [9]. Interestingly, dietary Ile reduced cathepsin B and L activities in hybrid bagrid catfish muscle. The increased fillet firmness, which is reflected by higher muscle shear force value, has also been addressed the importance of impacting fish quality [8, 63]. In the present study, optimal dietary Ile increased shear force value of fish muscle. Johnston et al. [63] reported that the fillet firmness could partly be reflected by muscle collagen content. The hydroxyproline was the major amino acid in collagen, which mainly constituted the extracellular structure of muscle. This study found the optimal dietary Ile level significantly increased hydroxyproline concentration in fish muscle, which was in line with grass carp [9]. Altogether, these results showed a benefit that dietary Ile could improve flesh quality by influencing muscle physicochemical characteristics.

Additionally, the texture characteristics, such as hardness, springiness, cohesiveness, gumminess, chewiness, and resilience, were important freshness factors in evaluating fish flesh quality [15]. The hardness is fundamental texture index reflecting the internal binding force of muscle [64]. Consumers tend not to choose firmer fillet due to the higher flesh hardness. The chewiness is defined as the energy required masticating and determined by firmness, cohesiveness, springiness and resilience [65, 66]. The present results indicated that springiness, cohesiveness, and resilience were significantly increased but chewiness was decreased in the muscle of hybrid bagrid catfish fed with increased dietary Ile. These results were similar to those reported in grass carp [9]. Accordingly, the present study for the first time showed dietary Ile existed a significant effect on muscle texture.

Fish muscle is easy to be subjected to oxidative stress, which results in the degenerative damage of flesh quality mainly by reducing muscle pH value, water holding capacity, and tenderness of fish [67, 68]. Previous studies have indicated that flesh quality was closely related to antioxidant capacity [3, 6]. In the present study, dietary Ile significantly decreased muscle ROS, MDA, and PC contents. This results were in accordance with previous report in grass carp [9]. The ASA and AHR activities are two critical indicators used to evaluate the total scavenging capacities of O^2−^ and OH^−^, respectively [49]. The current study presented that dietary Ile increased the AHR and ASA activities in the hybrid bagrid catfish muscle. Fish antioxidant system, as an important regulator to scavenge excess O^2−^ and OH^−^, was consisted of non-enzymatic compounds and antioxidant enzymes [50]. Meanwhile, the activities of antioxidant enzymes are tightly regulated by their corresponding genes [49]. The present study indicated that dietary Ile could increase T-SOD, CAT, GPX, GST, and GR activities as well as GSH content, depending on the up-regulated of *SOD*, *CAT*, *GPX1a*, *GST*, and *GCLC* mRNA expressions in the hybrid bagrid catfish muscle. In fish, studies have shown that Nrf2, which was inhibited by Keap1, played a critical role in regulating antioxidant enzyme-related genes expressions [3, 6]. Dietary Ile significantly increased *Nrf2* mRNA level but decreased *Keap1* mRNA level in muscle, suggesting that dietary Ile might improve muscle antioxidant gene expression by regulating the Keap1-Nrf2 pathway. In addition to being associated with antioxidant capacity, previous studies have reported a strong relationship between flesh quality and muscle growth [63, 66]. However, the detailed regulation mechanism of muscle growth associated with flesh quality need further research.

### Dietary Ile improved muscle growth of fish

The Ile, one of BCAA, have been reported the importance of effecting fish growth performance [9, 14, 59]. Fish growth is mainly determined by muscle growth, which constitutes the major edible sources of the fillet [6, 8]. The IGFs play an important role in promoting muscle growth by regulating myocyte hyperplasia, hypertrophy, and muscle protein accumulation [24, 28]. In fish, the IGFs, including IGF1 and IGF2, exert biological functions primarily through the binding and activation of the IGF1R, which are evolutionarily conserved genes compared to mammals [27]. In the present study, the higher mRNA levels of *IGF1*, *IGF2*, and *IGF1R* of hybrid bagrid catfish muscle was observed in fish fed 12.5 g/kg diet. Similar results have been reported in juvenile hybrid grouper (*Epinephelus fuscoguttatus♀ × Epinephelus lanceolatus♂*), where Ile significantly up-regulated liver *IGF-I* mRNA level [69]. Meanwhile, muscle *IGF1* (*r* = 0.821, *P* <  0.05) and *IGF2* (*r* = 0.750, *P* = 0.052) mRNA levels were positively correlated with *IGF1R* mRNA level, suggesting that IGF1 and IGF2 exerts its biological functions directly via binding to IGF1R. Similarly, studies in zebrafish reported that IGF1 and IGF2 bound to the IGF1R to promote muscle growth [70]. However, the specific mechanism of whether dietary Ile improve muscle growth via affecting IGFs remains further investigation.

#### Dietary Ile improved myofiber hyperplasia and hypertrophy

Fish muscle growth is constituted the myocytes’ proliferation and differentiation, which are sustained by myofiber hyperplasia and hypertrophy. Myofibers hyperplasia and hypertrophy could partly be reflected by the frequency of myofibers diameter [71]. The frequency of myofiber smaller than 20 μm indicated hyperplasia, that frequency of myofiber lager than 50 μm denoted hypertrophy [59, 72]. In the current study, the percentage of myofiber with diameters smaller than 20 μm and larger than 50 μm significantly rose in fish fed dietary Ile. However, a study in Nile tilapia juveniles showed dietary Ile exhibited a non-effect in the frequency of myofiber diameter [59]. The discrepancies results may be due to the differences in fish species, feeding management, and age. All the results above suggested that dietary Ile could improve muscle growth by improving myofiber hyperplasia and hypertrophy. However, the detailed mechanisms behind the effects of dietary Ile on muscle growth need further characterization.

Fish muscle growth is controlled by several myogenesis-transcription factors [6]. The PCNA is the necessary maker for myosatellite lineages activation; subsequently, Myod and Myf5 determine myoblast proliferation, whereas Myog and Mrf4 maintain the differentiation program [31, 73]. The MyHC is necessary for myofiber hyperplasia and hypertrophy [74]. Dietary Ile significantly increased the *PCNA*, *Myod*, *Myf5*, *Myog*, *Mrf4*, and *MyHC* mRNA levels in hybrid bagrid catfish muscle, indicating that dietary Ile could improve muscle development by increasing muscle cell proliferation and differentiation. These results agreed with mice myocyte, where suitable Ile concentration in BCAA could increase the mRNA levels of *Myog* and *Myod* [45]. The MSTN performed as a negative regulator of myoblasts proliferation [75]. In this study, dietary Ile decreased the *MSTN* mRNA level in hybrid bagrid catfish muscle. Similar results were reported in mice myocyte [45]. Overall, dietary Ile could improve muscle cell proliferation and differentiation program by regulating PCNA, MRFs, and MSTN expression might be partly attributed to enhance muscle growth.

#### Dietary Ile improved muscle protein deposition

Fish muscle growth is also closely associated with muscle protein content, which is determined by the balance between protein synthesis and degradation [27, 76]. In present result, dietary Ile significantly up-regulated muscle protein content. Similar results were observed in fingerling spotted snakehead [14], grass carp [9], and Nile tilapia [59]. Previous studies have observed that IGF1-PI3K-AKT acts a positive regulator in protein synthesis in mammals [77]. Upon phosphorylation activation by PI3K, AKT would improve protein synthesis processes via the TOR pathway [78–80]. In response to stimulation by the phosphorylation of AKT, TOR phosphorylates 4E-BP1 and S6K1, which are the main regulators in the initiation of protein translation and protein synthesis, respectively [81, 82]. Despite the fact that they have been studied in mammalian muscle growth, however, the regulatory role of amino acids in fish remains poorly explored. Previous studies indicated that dietary threonine and leucine could improve muscle protein synthesis by regulating the IGF1-PI3K-AKT-TOR pathway [23, 29]. In this framework, dietary Ile increased the expressions of *PI3K*, *AKT*, *TOR*, and *S6K1* in muscle. Whereas, the expression of *4E-BP1* showed a contrary trend to that of *S6K1*. Compared with control and Ile-excess diet, 12.5 g/kg diet increased phosphorylation of AKT, TOR, and S6K1. Correlation analysis showed that the protein content was positively correlated with *PI3K* (*r* = 0.607, *P* = 0.148), *AKT* (*r* = 0.847, *P* < 0.05), *TOR* (*r* = 0.786, *P* < 0.05), and *S6K1* (*r* = 0.929, *P* < 0.01) mRNA levels and negatively correlated with *4E-BP1* (*r* = − 0.714, *P* = 0.071) mRNA level, suggesting that dietary Ile could increase protein synthesis via AKT-TOR-S6K1 signaling pathway in hybrid bagrid catfish. These results were in line with the study in bovine [37] and goat [38] mammary tissue, and MAC-T cells [83], where Ile significantly increased the AKT, mTOR, and S6K1 protein phosphorylation levels. Gan et al. [9] also reported that appropriate dietary Ile increased *TOR* and *S6K1* expressions in grass carp muscle. These results indicated that the activation of the AKT-TOR-S6K1 signaling pathway by dietary Ile could improve fish muscle protein synthesis. However, more studies need to take a closer insight into the detailed mechanism in how dietary Ile improves fish muscle protein synthesis.

In fish muscle, protein degradation relies on three mechanisms, the autophagy-lysosome system, UPS, and calpain system [84], with the UPS as the primary way of proteolysis in nutrients deficiency [85]. The PI3K-AKT signaling pathway also play a central role in impeding protein degradation processes by inhibiting the activity of FOXO proteins inducing the nuclear export of these proteins through the nuclear pore complex [86]. The FOXO transcription factors, which induces protein degradation by increasing the expression of MURF-1 and MAFBX involved in the UPS pathway [33, 39]. Several reports in mammals have reported that Ile suppressed muscle protein degradation by inhibiting the UPS pathway [45, 87, 88]. A report in rainbow trout primary myocytes represented that dietary Ile declined the protein degradation rate [84]. In the present study, the mRNA expressions of *FOXO3a*, *MURF-1*, and *MAFBX* in fish muscle were declined with the dietary Ile levels up to 12.5 g/kg diet and increased thereafter. Compared with control and Ile-excess diets, 12.5 g/kg diet increased phosphorylation of FOXO3a, but it did decrease the protein abundances of MURF-1 and MAFBX, suggesting that dietary Ile inhibited the activity of FOXO3a and reduced the muscle protein degradation by the UPS pathway. Moreover, the negative correlations among the protein content and mRNA expressions of *FOXO3a* (*r* = − 0.332; *P* = 0.467), *MURF-1* (*r* = − 0.633; *P* = 0.127), and *MAFBX* (*r* = − 0.589; *P* = 0.164) have been observed in this study. These results suggested that dietary Ile impeded protein degradation was partly related to AKT-FOXO3a-MURF-1/MAFBX pathway. To our best knowledge, this is the first to find that dietary Ile could increase the phosphorylation of FOXO3a, but decrease MURF-1 and MAFBX expressions in fish muscle resulting in reduced protein degradation. Collectively, the above results further sustained the conclusion that dietary Ile improved muscle growth might be partly attributed to the AKT-FOXO3a signaling in fish.

## Conclusions

In conclusion, this study clarified that dietary Ile was contributed to the flesh quality of hybrid bagrid catfish. Meanwhile, the data for the first time indicated that suitable dietary Ile increased muscle mass and influenced myofiber diameter distribution, which might be through promoting protein synthesis by activating of the AKT-TOR-S6K1 signaling pathway, and inhibiting protein degradation by altering AKT-FOXO3a-MURF-1/MAFBX abundances during an 8-week growth period. Finally, based on flesh quality (chewiness and ROS) and protein deposition (protein content) related parameters, dietary Ile levels for hybrid bagrid catfish were estimated to be 14.19, 12.36, and 12.78 g/kg diet, corresponding to 36.59, 31.87, and 32.96 g/kg dietary protein, respectively.

## Supplementary Information

**Additional file 1: Table S1.** Composition and nutrient content of basal diet, g/kg. **Table S2.** The primers and annealing temperature (AT) used for real-time quantitative PCR. ** Fig. S1**. The final body weight (**a**), feed intake (**b**), percent weight gain (**c**), specific growth rate (**d**), protein efficiency ratio (**e**), and feed efficiency (**f**) of hybrid bagrid catfish fed diets with graded levels of Ile for 8 weeks. Data represent means ± SEM, of three replicates. Values having different letters are significantly different (*P* < 0.05).

## Data Availability

The datasets produced and/or analyzed during the current study are available from the corresponding author on reasonable request.

## Abbreviations

4E-BP1: eIF4E-binding protein 1
AKT: Protein kinase B
BCAA: Branched-chain amino acids
ECL: Chemoluminescence
eIF4E: Eukaryotic translation initiation factor 4E
FOXO3a: Forkhead box protein O3a
IGF-1: Insulin-like growth factor 1
IGF-2: Insulin-like growth factor 2
IGF-1R: Insulin-like growth factor 1 receptor
Ile: Isoleucine
Keap1: Kelch-like ECH-associated protein 1
MAFBX: Muscle atrophy F box
MDA: Malondialdehyde
Mrf4: Myogenic regulatory factor 4
MRFs: Myogenic regulatory factors
Nrf2: Nuclear factor erythroid 2-related factor 2
MSTN: Myostatin
MURF-1: Muscle-specific RING-finger 1
Myf5: Myogenic factor 5
Myod: Myogenic differentiation
Myog: Myogenin
PC: Protein carbonyl
PCNA: Proliferating cell nuclear antigen
PI3K: Phosphoinositide 3-kinase
PVDF: Polyvinylidene fluoride
ROS: Reactive oxygen species
RT-PCR: Real-time polymerase chain reaction
S6K1: Ribosomal protein S6 kinase 1
TOR: Target of rapamycin
TPA: Texture profile analysis
UPS: Ubiquitin-proteasome system

## References

[1] Publishing Group in FAO's Office for Corporate Communication (2020). FAO yearbook: Fishery and aquaculture statistics.

[2] Grunert KG, Bredahl L, Brunso K (2004). Consumer perception of meat quality and implications for product development in the meat sector-a review. Meat Ence.

[3] Jiang W, Chen L, Liu Y, Feng L, Wu P, Jiang J (2019). Impact and consequences of dietary riboflavin deficiency treatment on flesh quality loss in on-growing grass carp (*Ctenopharyngodon idella*). Food Funct.

[4] Gisbert E, Mozanzadeh MT, Kotzamanis Y, Estévez A (2016). Weaning wild flathead grey mullet (*Mugil cephalus*) fry with diets with different levels of fish meal substitution. Aquaculture..

[5] Wu C, Ye J, Chen L, Lu Z (2016). The effects of dietary carbohydrate on the growth, antioxidant capacities, innate immune responses and pathogen resistance of juvenile black carp *Mylopharyngodon piceus*. Fish Shellfish Immun.

[6] Zhao Y, Li J, Yin L, Feng L, Liu Y, Jiang W (2019). Effects of dietary glutamate supplementation on flesh quality, antioxidant defense and gene expression related to lipid metabolism and myogenic regulation in Jian carp (*Cyprinus carpio* var. Jian). Aquaculture.

[7] Zhao H, Xia J, Zhang X, He X, Li L, Tang R (2018). Diet affects muscle quality and growth traits of grass carp (*Ctenopharyngodon idellus*): a comparison between grass and artificial feed. Front Physiol.

[8] Wu P, Qu B, Feng L, Jiang W, Kuang S, Jiang J (2020). Dietary histidine deficiency induced flesh quality loss associated with changes in muscle nutritive composition, antioxidant capacity, Nrf2 and TOR signaling molecules in on-growing grass carp (*Ctenopharyngodon idella*). Aquaculture.

[9] Gan L, Jiang W, Wu P, Liu Y, Jiang J, Li S (2014). Flesh quality loss in response to dietary isoleucine deficiency and excess in fish: a link to impaired Nrf2-dependent antioxidant defense in muscle. PLoS One.

[10] Zhao Y, Yan M, Jiang Q, Yin L, Zhou X, Feng L (2021). Isoleucine improved growth performance, and intestinal immunological and physical barrier function of hybrid catfish *Pelteobagrus vachelli× Leiocassis longirostris*. Fish Shellfish Immun.

[11] Yin L, Zhao Y, Zhou X, Yang C, Feng L, Liu Y (2020). Effect of dietary isoleucine on skin mucus barrier and epithelial physical barrier functions of hybrid bagrid catfish *Pelteobagrus vachelli× Leiocassis longirostris*. Fish Physiol Biochem.

[12] Fuentes A, Fernández-Segovia I, Serra JA, Barat JM (2010). Comparison of wild and cultured sea bass (*Dicentrarchus labrax*) quality. Food Chem.

[13] Kim YHB, Warner RD, Rosenvold K (2014). Influence of high pre-rigor temperature and fast pH fall on muscle proteins and meat quality: a review. Anim Prod Sci.

[14] Sharf Y, Khan MA (2020). Effect of dietary isoleucine level on growth, protein retention efficiency, haematological parameter, lysozyme activity and serum antioxidant status of fingerling *Channa punctatus* (Bloch). Aquac Nutr.

[15] Moreno HM, Montero MP, Gomez-Guilleen MC, Fernaendez-Martien F, Morkere T, Borderieas J (2012). Collagen characteristics of farmed Atlantic salmon with firm and soft fillet texture. Food Chem.

[16] Vacha F, Cepak M, Urbanek M, Vejsada P, Hartvich P, Rost M (2013). Impact of long-term storage on the instrumental textural properties of frozen common carp (*Cyprinus carpio*, L.) flesh. Int J Food Prop.

[17] Periago MJ, Ayala MD, López-Albors O, Abdel I, Martínez C, García-Alcázar A (2005). Muscle cellularity and flesh quality of wild and farmed sea bass, *Dicentrarchus labrax* L. Aquaculture..

[18] Alami-Durante H, Bazin D, Cluzeaud M, Fontagné-Dicharry S, Kaushik S, Geurden I (2018). Effect of dietary methionine level on muscle growth mechanisms in juvenile rainbow trout (*Oncorhynchus mykiss*). Aquaculture..

[19] Aguiar A, Vechetti-Júnior I, Alves DSR, Castan E, Milanezi-Aguiar R, Padovani C (2012). Myogenin, MyoD and IGF-I regulate muscle mass but not fiber-type conversion during resistance training in rats. Int J Sports Med.

[20] Johnston IA, Lee H, Macqueen DJ, Paranthaman K, Kawashima C, Anwar A (2009). Embryonic temperature affects muscle fibre recruitment in adult zebrafish: genome-wide changes in gene and microRNA expression associated with the transition from hyperplastic to hypertrophic growth phenotypes. J Exp Biol.

[21] Duan C, Ren H, Gao S (2010). Insulin-like growth factors (IGFs), IGF receptors, and IGF-binding proteins: roles in skeletal muscle growth and differentiation. Gen Com Endocr.

[22] Kawanago M, Takemura S, Ishizuka R, Shioya I (2015). Effects of dietary branched-chain amino acid supplementation on growth and skeletal muscle cellularity in Japanese amberjack *Seriola quinqueradiata*. Fisheries Sci.

[23] Zhao Y, Jiang Q, Zhou X, Xu S, Feng L, Liu Y (2020). Effect of dietary threonine on growth performance and muscle growth, protein synthesis and antioxidant-related signalling pathways of hybrid catfish *Pelteobagrus vachelli♀× Leiocassis longirostris♂*. Brit J Nutr..

[24] Azizi S, Nematollahi MA, Amiri BM, Vélez EJ, Gutiérrez J (2016). IGF-I and IGF-II effects on local IGF system and signaling pathways in gilthead sea bream (*Sparus aurata*) cultured myocytes. Gen Comp Endocrinol.

[25] Mohamed RA, Elbialy ZI, Abd El Latif AS, Shukry M, Assar DH, El Nokrashy AM (2020). Dietary clenbuterol modifies the expression of genes involved in the regulation of lipid metabolism and growth in the liver, skeletal muscle, and adipose tissue of Nile tilapia (*Oreochromis niloticus*). Aquacult Rep.

[26] Lee S, McPherron AC (2001). Regulation of myostatin activity and muscle growth. P Natl Acad Sci USA.

[27] Fuentes EN, Valdés JA, Molina A, Bj Rnsson BRT (2013). Regulation of skeletal muscle growth in fish by the growth hormone-insulin-like growth factor system. Gen Com Endocr..

[28] Codina M, Montserrat N, Garcia De La Serrana D, Ralliere C, Gabillard J, Navarro I (2006). Role of insulin and IGFs in fish muscle development and quality. Arch Anim Breed.

[29] Zhao Y, Li J, Jiang Q, Zhou X, Feng L, Liu Y (2020). Leucine improved growth performance, muscle growth, and muscle protein deposition through AKT/TOR and AKT/FOXO3a signaling pathways in hybrid catfish *pelteobagrus vachelli × Leiocassis longirostris*. Cells (Basel).

[30] NRC (2011). Nutrient requirements of fish and shrimp.

[31] Johnston IA, Bower NI, Macqueen DJ (2011). Growth and the regulation of myotomal muscle mass in teleost fish. J Exp Biol.

[32] Fuentes EN, Björnsson BT, Valdés JA, Einarsdottir IE, Lorca B, Alvarez M (2011). IGF-I/PI3K/AKT and IGF-I/MAPK/ERK pathways in vivo in skeletal muscle are regulated by nutrition and contribute to somatic growth in the fine flounder. Am J Physiol Reg, Int Com Physiol.

[33] Glass DJ (2010). Signaling pathways perturbing muscle mass. Curr Opin Clin Nutr Metab Care.

[34] Léger B, Cartoni R, Praz M, Lamon S, Dériaz O, Crettenand A (2006). Akt signalling through GSK-3beta, mTOR and Foxo1 is involved in human skeletal muscle hypertrophy and atrophy. J Physiol.

[35] Proud CG (2007). Amino acids and mTOR signalling in anabolic function. Biochem Soc T.

[36] Gao Y, Lu S, Wu M, Yao W, Wu X (2019). Effects of dietary protein levels on growth, feed utilization and expression of growth related genes of juvenile giant grouper (*Epinephelus lanceolatus*). Aquaculture.

[37] Apelo SA, Singer LM, Lin XY, McGilliard ML, St-Pierre NR, Hanigan MD (2014). Isoleucine, leucine, methionine, and threonine effects on mammalian target of rapamycin signaling in mammary tissue. J Dairy Sci.

[38] Xu LB, Hanigan MD, Lin XY, Li MM, Wang ZH (2019). Effects of jugular infusions of isoleucine, leucine, methionine, threonine, and other amino acids on insulin and glucagon concentrations, mammalian target of rapamycin (mTOR) signaling, and lactational performance in goats. J Dairy Sci.

[39] Zeitz JO, Käding S, Niewalda IR, Most E, Dorigam JCDP, Eder K (2019). The influence of dietary leucine above recommendations and fixed ratios to isoleucine and valine on muscle protein synthesis and degradation pathways in broilers. Poultry Sci.

[40] Brunet A, Bonni A, Zigmond MJ, Lin MZ, Juo P, Hu LS (1999). Akt promotes cell survival by phosphorylating and inhibiting a forkhead transcription factor. Cell..

[41] Von Haehling S, Ebner N, Dos Santos MR, Springer J, Anker SD (2017). Muscle wasting and cachexia in heart failure: mechanisms and therapies. Nat Rev Cardiol.

[42] White JP, Gao S, Puppa MJ, Sato S, Welle SL, Carson JA (2013). Testosterone regulation of Akt/mTORC1/FoxO3a signaling in skeletal muscle. Mol Cell Endocrinol.

[43] Stitt TN, Drujan D, Clarke BA, Panaro F, Timofeyva Y, Kline WO (2004). The IGF-1/PI3K/Akt pathway prevents expression of muscle atrophy-induced ubiquitin ligases by inhibiting FOXO transcription factors. Mol Cell.

[44] de Palma L, Marinelli M, Pavan M, Orazi A (2008). Ubiquitin ligases MuRF1 and MAFbx in human skeletal muscle atrophy. Joint Bone Spine.

[45] Duan Y, Zeng L, Li F, Wang W, Li Y, Guo Q (2017). Effect of branched-chain amino acid ratio on the proliferation, differentiation, and expression levels of key regulators involved in protein metabolism of myocytes. Nutrition..

[46] Zheng L, Wei H, He P, Zhao S, Xiang Q, Pang J (2017). Effects of supplementation of branched-chain amino acids to reduced-protein diet on skeletal muscle protein synthesis and degradation in the fed and fasted states in a piglet model. Nutrients..

[47] Fishery Bureau of the Ministry of Agriculture (2019). China Fishery Statistical Yearbook 2004-2019.

[48] Bohne VJB, Hamre K, Arukwe A (2007). Hepatic metabolism, phase I and II biotransformation enzymes in Atlantic salmon (*Salmo Salar*, L) during a 12 week feeding period with graded levels of the synthetic antioxidant, ethoxyquin. Food Chem Toxicol.

[49] Jiang J, Wu X, Zhou X, Feng L, Liu Y, Jiang W (2016). Glutamate ameliorates copper-induced oxidative injury by regulating antioxidant defences in fish intestine. Brit J Nutr.

[50] Liu X, Feng L, Jiang W, Wu P, Jiang J, Yang D (2020). (2-Carboxyethyl) dimethylsulfonium Bromide (Br-DMPT) improves muscle flesh quality and antioxidant status of on-growing grass carp (*Ctenopharyngodon idella*) fed non-fish meal diets. Aquaculture.

[51] Brinker A, Reiter R (2011). Fish meal replacement by plant protein substitution and guar gum addition in trout feed, part I: effects on feed utilization and fish quality. Aquaculture..

[52] LeBel CP, Ischiropoulos H, Bondy SC (1992). Evaluation of the probe 2′, 7′-dichlorofluorescin as an indicator of reactive oxygen species formation and oxidative stress. Chem Res Toxicol.

[53] Zhang X, Wang J, Tang R, He X, Li L, Takagi Y (2019). Improvement of muscle quality of grass carp (*Ctenopharyngodon idellus*) with a bio-floating bed in culture ponds. Front Physiol.

[54] Dogra C, Changotra H, Wergedal JE, Kumar A (2006). Regulation of phosphatidylinositol 3-kinase (PI3K)/Akt and nuclear factor-kappa B signaling pathways in dystrophin-deficient skeletal muscle in response to mechanical stretch. J Cell Physiol.

[55] de Almeida FLA, Carvalho RF, Pinhal D, Padovani CR, Martins C, Dal P-SM (2008). Differential expression of myogenic regulatory factor MyoD in pacu skeletal muscle (*Piaractus mesopotamicus* Holmberg 1887: Serrasalminae, Characidae, Teleostei) during juvenile and adult growth phases. Micron.

[56] Livak KJ, Schmittgen TD (2001). Analysis of relative gene expression data using real-time quantitative PCR and the 2- ΔΔCT method. Methods..

[57] Vandesompele J, De Preter K, Pattyn F, Poppe B, Van Roy N, De Paepe A (2002). Accurate normalization of real-time quantitative RT-PCR data by geometric averaging of multiple internal control genes. Genome Biol.

[58] Perez-Velazquez M, Gatlin DM, González-Félix ML, García-Ortega A, de Cruz CR, Juárez-Gómez ML (2019). Effect of fishmeal and fish oil replacement by algal meals on biological performance and fatty acid profile of hybrid striped bass (*Morone crhysops ♀ × M. Saxatilis ♂*). Aquaculture.

[59] Neu DH, Boscol WR, Almeida FLAD, Zaminhan HM, Dallagnol JM, Furuya WM (2017). Growth performance, hematology, and muscle growth in isoleucine fed nile tilapia. Bol Inst Pesca.

[60] Grigorakis K (2007). Compositional and organoleptic quality of farmed and wild gilthead sea bream (*Sparus aurata*) and sea bass (*Dicentrarchus labrax*) and factors affecting it: a review. Aquaculture..

[61] Li T, Hu W, Li J, Zhang X, Zhu J, Li X (2012). Coating effects of tea polyphenol and rosemary extract combined with chitosan on the storage quality of large yellow croaker (*Pseudosciaena crocea*). Food Control.

[62] Bahuaud D, Morkore T, Ostbye TK, Veiseth-Kent E, Thomassen MS, Ofstad R (2010). Muscle structure responses and lysosomal cathepsins B and L in farmed Atlantic salmon (*Salmo salar* L.) pre- and post-rigor fillets exposed to short and long-term crowding stress. Food Chem.

[63] Johnston IA, Li X, Vieira VLA, Nickell D, Dingwall A, Alderson R (2006). Muscle and flesh quality traits in wild and farmed Atlantic salmon. Aquaculture..

[64] Li H, Pan Y, Liu L, Li Y, Huang X, Zhong Y (2019). Effects of high-fat diet on muscle textural properties, antioxidant status and autophagy of Chinese soft-shelled turtle (*Pelodiscus sinensis*). Aquaculture..

[65] Mitchell J (2003). Food texture and viscosity: concept and measurement. Int J Food Sci Tech.

[66] Song D, Yun Y, Mi J, Luo J, Jin M, Nie G (2020). Effects of faba bean on growth performance and fillet texture of Yellow River carp, *Cyprinus carpio haematopterus*. Aquacult Rep.

[67] Falowo AB, Fayemi PO, Muchenje V (2014). Natural antioxidants against lipid-protein oxidative deterioration in meat and meat products: a review. Food Res Int.

[68] Morachis-Valdez G, Dublán-García O, López-Martínez LX, Galar-Martínez M, Saucedo-Vence K, Gómez-Oliván LM (2015). Chronic exposure to pollutants in Madín reservoir (Mexico) alters oxidative stress status and flesh quality in the common carp *Cyprinus carpio*. Environ Sci Pollut R.

[69] Zhou Z, Wu X, Li X, Dong Y, Zhang Y (2020). The optimum dietary isoleucine requirement of juvenile hybrid grouper (*Epinephelus fuscoguttatus ♀ × Epinephelus lanceolatus ♂*). Aquac Nutr.

[70] Pozios KC, Ding J, Degger B, Upton Z, Duan C (2001). IGFs stimulate zebrafish cell proliferation by activating MAP kinase and PI3-kinase-signaling pathways. Am J Physiol Reg Int Com Physiol.

[71] Valente LMP, Moutou KA, Concei OLEC, Engrola S, Fernandes JMO, Johnston IA (2013). What determines growth potential and juvenile quality of farmed fish species?. Rev Aquacult.

[72] Valente LMP, Rocha E, Gomes EFS, Silva MW, Fauconneau B (2010). Growth dynamics of white and red muscle fibres in fast- and slow-growing strains of rainbow trout. J Fish Biol.

[73] Zanou N, Gailly P (2013). Skeletal muscle hypertrophy and regeneration: interplay between the myogenic regulatory factors (MRFs) and insulin-like growth factors (IGFs) pathways. Cell Mol Life Ences.

[74] Biga PR, Goetz FW (2006). Zebrafish and giant danio as models for muscle growth: determinate vs. indeterminate growth as determined by morphometric analysis. Am J Physiol Regul Integr Comp Physiol.

[75] Seiliez I, Sabin N, Gabillard JC (2012). Myostatin inhibits proliferation but not differentiation of trout myoblasts. Mol Cell Endocr.

[76] Adams GR, Haddad F (1997). The relationships among IGF-1, DNA content, and protein accumulation during skeletal muscle hypertrophy. J Appl Physiol.

[77] Schiaffino S, Dyar KA, Ciciliot S, Blaauw B, Sandri M (2013). Mechanisms regulating skeletal muscle growth and atrophy. FEBS J.

[78] Cantley LC (2002). The phosphoinositide 3-kinase pathway. Science..

[79] Fan J, Kou X, Jia S, Yang X, Yang Y, Chen N (2016). Autophagy as a potential target for sarcopenia. J Cell Physiol.

[80] Xu Y, Li N, Xiang R, Sun P (2014). Emerging roles of the p38 MAPK and PI3K/AKT/mTOR pathways in oncogene-induced senescence. Trends Biochem Sci.

[81] Jewell JL, Kim YC, Russell RC, Yu FX, Park HW, Plouffe SW (2015). Differential regulation of mTORC1 by leucine and glutamine. Ence..

[82] Bodine SC, Stitt TN, Gonzalez M, Kline WO, Stover GL, Bauerlein R (2001). AKT/mTOR pathway is a crucial regulator of skeletal muscle hypertrophy and can prevent muscle atrophy in vivo. Nat Cell Biol.

[83] Appuhamy JADR, Knoebel NA, Nayananjalie WAD, Escobar J, Hanigan MD (2012). Isoleucine and leucine independently regulate mTOR signaling and protein synthesis in MAC-T cells and bovine mammary tissue slices. J Nutr.

[84] Cleveland BM, Radler LM (2019). Essential amino acids exhibit variable effects on protein degradation in rainbow trout (*Oncorhynchus mykiss*) primary myocytes. Com Biochem Physiol A.

[85] Cleveland BM, Kenney PB, Manor ML, Weber GM (2012). Effects of feeding level and sexual maturation on carcass and fillet characteristics and indices of protein degradation in rainbow trout (*Oncorhynchus mykiss*). Aquaculture.

[86] Huang H, Tindall DJ (2011). Regulation of FOXO protein stability via ubiquitination and proteasome degradation. BBA-Mol Cell Res.

[87] Zeitz JO, Käding S, Niewalda IR, Machander V, de Paula Dorigam JC, Eder K (2019). Effects of leucine supplementation on muscle protein synthesis and degradation pathways in broilers at constant dietary concentrations of isoleucine and valine. Arch Anim Nutr.

[88] Yamanashi K, Kinugawa S, Fukushima A, Kakutani N, Takada S, Obata Y (2020). Branched-chain amino acid supplementation ameliorates angiotensin II-induced skeletal muscle atrophy. Life Sci.

