# Supplementary material for: Dietary isoleucine improved flesh quality, muscle antioxidant capacity, and muscle growth associated with AKT/TOR/S6K1 and AKT/FOXO3a signaling in hybrid bagrid catfish (Pelteobagrus vachelli♀ × Leiocassis longirostris♂)
Source: J Anim Sci Biotechnol. 2021 Apr 19;12:53. doi: 10.1186/s40104-021-00572-4 (PMC8054373; doi:10.1186/s40104-021-00572-4)
Supplement: Supplementary file 1 — Additional file 1: Table S1. Composition and nutrient content of basal diet, g/kg. Table S2. The primers and annealing temperature (AT) used for real-time quantitative PCR. Fig. S1. The final body weight (a), feed intake (b), percent weight gain (c), specific growth rate (d), protein efficiency ratio (e), and feed efficiency (f) of hybrid bagrid catfish fed diets with graded levels of Ile for 8 weeks. Data represent means ± SEM, of three replicates. Values having different letters are significantly different (P < 0.05). [file 40104_2021_572_MOESM1_ESM.docx]

**Table S1** Composition and nutrient content of basal diet, g/kg.

| Ingredients |  | Nutrients content^e^ |  |
| --- | --- | --- | --- |
| Fish meal | 170.0 | Crude protein | 387.8 |
| Casein | 10.0 | Crude lipid | 71.0 |
| Gelatin | 10.0 | Crude ash | 145.2 |
| Corn starch | 168.3 | ω-3 | 15.2 |
| α-Starch | 30.0 | ω-6 | 13.9 |
| Fish oil | 26.0 | Available phosphorus | 14.8 |
| Soybean oil | 210.0 |  |  |
| Monocalcium phosphate | 40.0 |  |  |
| Vitamin premix ^a^ | 10.0 |  |  |
| Ile premix ^b^ | 120.0 |  |  |
| Mineral element premix ^c^ | 20.0 |  |  |
| Crystal amino acid premix ^d^ | 170.0 |  |  |
| Choline chloride (50%) | 10.0 |  |  |
| Ethoxy quinoline (30%) | 0.5 |  |  |
| Cellulose | 5.0 |  |  |
| Xanthophy II | 0.2 |  |  |
| Total | 1000.0 |  |  |

^a^ The premix provides vitamin for a kilogram of diet: *D, L*-α-tocopherol acetate (500 g/kg), 53.600 g; menadione (230 g/kg), 0.217 g; retinyl acetate (500,000 IU/g), 8.063 g; cholecalciferol (500,000 IU/g), 0.100 g; thiamin nitrate (900 g/kg), 0.111 g; riboflavine (800 g/kg), 1.125 g; pyridoxine hydrochloride (810 g/kg), 0.370 g; cyanocobalamin (10 g/kg), 0.100 g; niacin (990 g/kg), 3.143 g; folic acid (960 g/kg), 0.521 g; meso-inositol (990 g/kg), 52.323 g; D-biotin(20 g/kg), 5.0 g; calcium-*D*-pantothenate (900 g/kg), 1.667 g; ascorhyl acetate (930 g/kg), 86.022 g. All ingredients were diluted with corn starch to 1 kg.

^b^ The premix provides Ile, glycine, and corn starch for a kilogram of diet 1 to 7: *L*-Ile 0.000, 20.833, 41.667, 62.500, 83.333, 104.167, 125.000 g; glycine 89.777, 74.814, 59.851, 44.888, 29.926, 14.963, 0.000 g; corn starch 910.223, 904.353, 898.482, 892.612, 886.741, 880.871, 875.000 g, respectively.

^c^ The premix provides mineral for a kilogram of diet: FeSO_4_·7H_2_O (300 g/kg Fe), 13.333 g; CuSO_4_·5H_2_O (250 g/kg Cu), 1.300 g; ZnSO_4_·7H_2_O (345 g/kg Zn), 13.043 g; MnSO_4_·H_2_O (318 g/kg Mn), 4.717 g; KI (38 g/kg I), 1.447 g; NaSeO_3_ (10 g/kg Se), 1.000 g. All ingredients were diluted with CaCO_3_ to 1 kg.

^d^ The premix provides crystal amino acid for a kilogram of diet: lysine (780 g/kg), 125.204 g; methionine (990 g/kg), 60.071 g; tryptophan (980 g/kg), 11.723 g; arginine (997 g/kg), 116.491 g; histidine (997 g/kg), 27.508 g; threonine (980 g/kg), 163.764 g; leucine (985 g/kg), 20.824 g; phenylalanine (996 g/kg), 17.364 g; valine (992 g/kg), 11.302 g; cystine (999 g/kg), 11.432 g. All ingredients were diluted with corn starch to 1 kg.

^e^ Crude protein, crude fat, crude ash, and Ile contents were measured. Available phosphorus, ω-3 and ω-6 contents calculated according to NRC (2011).

**Table S2** The primers and annealing temperature (AT) used for real-time quantitative PCR.

| Gene name | Primer sequences (5′→3′) | AT, °C | GenBank ID |
| --- | --- | --- | --- |
| *CuZnSOD*-QF | TCACTTCAACCCCCACAACA | 63.3 | KX455916.1 |
| *CuZnSOD*-QR | CGGCAGTCACATTACCCAGAT |  |  |
| *CAT*-QF | ACACCGATGAGGGAAACTGG | 58.0 | KX455919 |
| *CAT*-QR | GTGGATGAAGGACGGGAACA |  |  |
| *GPX1a*-QF | GTGAATGGGAAAGACGCTC | 61.7 | MG773203 |
| *GPX1a*-QR | GCACACAGGACTCCAGATGA |  |  |
| *GST*-QF | CGGATGGGAAATGGAACG | 58.0 | XM_027154055.1 |
| *GST*-QR | GGATAATGCTCCTGACTCAACC |  |  |
| *GCLC*-QF | GACAAACGGAGGAAGGAGG | 58.2 | KX455918 |
| *GCLC*-QR | TCATCAGGAAAGAAGAGGGACT |  |  |
| *Keap1*-QF | GCATCCTCTTCACCTGTCT | 61.7 | MG773201 |
| *Keap1*-QR | CGTGTAGGCGAACTCTATC |  |  |
| *Nrf2*-QF | CGGAACAAGATGGAGAAGCC | 64.0 | KX455917 |
| *Nrf2*-QR | ACAGGGAGGAATGGAGGGA |  |  |
| *IGF1*-QF | ATCTGGGTAATGTGACTGCCGA | 56.8 | KX434878 |
| *IGF1*-QR | TTCATCATCTCCGCCCTTGC |  |  |
| *IGF2*-QF | GTGGAGGAATGCTGTTTTCGGAG | 61.4 | JN378897 |
| *IGF2*-QR | AACTTTCTGGAGCGGAGGATGG |  |  |
| *IGF1R*-QF | ACACCGATGAGGGAAACTGG | 56.6 | MG773202 |
| *IGF1R*-QR | GTGGATGAAGGACGGGAACA |  |  |
| *PCNA*-QF | GTTGATGGACTTGGATGTGGA | 60.1 | MK281343 |
| *PCNA*-QR | CGTTGCTGGTTTGGGAGA |  |  |
| *Myf5*-QF | CTCCAGTCCTTCATCATCCACC | 64.9 | MK253547 |
| *Myf5*-QR | CACTCGCACTCTGACCTTCGT |  |  |
| *Myod*-QF | CCTAATCAGAGGCTTCCCA | 55.5 | HM363525 |
| *Myod*-QR | TCACCGCTGTATTGTTCCA |  |  |
| *Myog*-QF | TACTTTTTCCCCGAACAGC | 57.6 | HQ246723 |
| *Myog*-QR | TCCAGTCCTACATTGCCAGA |  |  |
| *Mrf4*-QF | CAGACTGTCAGAGGACGGGG | 52.8 | MK281342 |
| *Mrf4*-QR | CAGCCTTCTCTTTGGTGGGA |  |  |
| *MyHC*-QF | GCAATGAAGGAGAACTATG | 60.0 | MK440319 |
| *MyHC*-QR | TCACACTTTCCTCAGCGT |  |  |
| *MSTN*-QF | ACGCCACTACCGAGACCG | 64.6 | DQ767967 |
| *MSTN*-QR | CTCAATACCCCAGTTTGTTTCC |  |  |
| *PI3K*-QF | GTGAATGGGAAAGACGCT | 62.6 | MG773208 |
| *PI3K*-QR | GCACACAGGACTCCAGATGA |  |  |
| *AKT*-QF | ACACGACCGCTTGTGCTTC | 61.7 | KX131157.1 |
| *AKT*-QR | TCCGTCCGTTATGCCCTCT |  |  |
| *TOR*-QF | GACAAACGGAGGAAGGAGG | 58.2 | MG773199 |
| *TOR*-QR | TCATCAGGAAAGAAGAGGGACT |  |  |
| *S6K1*-QF | GCAAACTGAATCTCCCACCC | 61.7 | MG773195 |
| *S6K1*-QR | AGGCTTGAAAGGCGGCTC |  |  |
| *4E-BP1*-QF | ACGCCACCCAGTTGCCTA | 62.6 | MG773207 |
| *4E-BP1*-QR | GGATGCTTTTGCTGCCGAC |  |  |
| *FOXO3a*-QF | GACTTCCGCTCTCGCACTAA | 60.5 | MK562423 |
| *FOXO3a*-QR | ATCATCAGCAACCTCATCCACT |  |  |
| *MAFBX*-QF | AACCTCTGTCACTACCACTTCACT | 54.8 | MK812970 |
| *MAFBX*-QR | GGTCGCTGTACTGCTCTTTATG |  |  |
| *MURF-1*-QF | CCGTTTTGAGGTGGTGCT | 53.6 | MK756118 |
| *MURF-1*-QR | TGTTCTCCAGTTGTTGCTTGTA |  |  |
| *18S*-QF | CCTGAGAAACGGCTACCACATCC | 57.1 | KP938527 |
| *18S*-QR | AGCAACTTTAATATACGCTATTGGAG |  |  |
| *β-actin*-QF | CCTAAAGCCAACAGGGAAAA | 59.0 | EU161066 |
| *β-actin*-QR | ATGGGGCAGAGCATAACC |  |  |

**Fig. S1** The final body weight (**a**), feed intake (**b**), percent weight gain (**c**), specific growth rate (**d**), protein efficiency ratio (**e**), and feed efficiency (**f**) of hybrid bagrid catfish fed diets with graded levels of Ile for 8 weeks. Data represent means ± SEM, of three replicates. Values having different letters are significantly different (*P* < 0.05).

**f**

**e**
